# Supplementary material for: LTA4H extensively associates with mRNAs and lncRNAs indicative of its novel regulatory targets
Source: PeerJ. 2023 Mar 10;11:e14875. doi: 10.7717/peerj.14875 (PMC10010175; doi:10.7717/peerj.14875)
Supplement: File S2 [file peerj-11-14875-s005.zip › Raw data files/Motif_results/cims/LTA4H_IP_1_vs_LTA4H_input_1/homerResults.html]

.// - Homer de novo Motif Results


# Homer *de novo* Motif Results (.//)

Known Motif Enrichment Results  
Gene Ontology Enrichment Results  
If Homer is having trouble matching a motif to a known motif, try copy/pasting the matrix file into
STAMP  
More information on motif finding results: HOMER
| Description of Results
| Tips
  
Total target sequences = 59547  
Total background sequences = 450107  
\* - possible false positive  

|  |  |  |  |  |  |  |  |  |
| --- | --- | --- | --- | --- | --- | --- | --- | --- |
| Rank | Motif | P-value | log P-pvalue | % of Targets | % of Background | STD(Bg STD) | Best Match/Details | Motif File |
| 1 |  | 1e-675 | -1.555e+03 | 4.83% | 1.19% | 10.2bp (24.1bp) | hsa-miR-4455 MIMAT0018977 Homo sapiens miR-4455 Targets (miRBase)(0.744) More Information \| Similar Motifs Found | motif file (matrix) |
| 2 |  | 1e-600 | -1.382e+03 | 10.20% | 4.58% | 6.3bp (20.9bp) | hsa-miR-3613-3p MIMAT0017991 Homo sapiens miR-3613-3p Targets (miRBase)(0.776) More Information \| Similar Motifs Found | motif file (matrix) |
| 3 |  | 1e-297 | -6.854e+02 | 20.21% | 14.26% | 8.7bp (16.4bp) | hsa-miR-4297 MIMAT0016846 Homo sapiens miR-4297 Targets (miRBase)(0.649) More Information \| Similar Motifs Found | motif file (matrix) |
| 4 |  | 1e-178 | -4.112e+02 | 2.20% | 0.81% | 9.6bp (21.0bp) | hsa-miR-4644 MIMAT0019704 Homo sapiens miR-4644 Targets (miRBase)(0.777) More Information \| Similar Motifs Found | motif file (matrix) |
| 5 |  | 1e-129 | -2.972e+02 | 2.21% | 0.97% | 9.8bp (14.6bp) | hsa-miR-432\* MIMAT0002815 Homo sapiens miR-432\* Targets (miRBase)(0.725) More Information \| Similar Motifs Found | motif file (matrix) |
| 6 |  | 1e-103 | -2.372e+02 | 18.34% | 14.88% | 9.8bp (16.2bp) | hsa-miR-4516 MIMAT0019053 Homo sapiens miR-4516 Targets (miRBase)(0.686) More Information \| Similar Motifs Found | motif file (matrix) |
| 7 |  | 1e-58 | -1.355e+02 | 0.14% | 0.00% | 8.9bp (11.1bp) | hsa-miR-3673 MIMAT0018096 Homo sapiens miR-3673 Targets (miRBase)(0.798) More Information \| Similar Motifs Found | motif file (matrix) |
| 8 |  | 1e-58 | -1.343e+02 | 0.92% | 0.39% | 7.8bp (13.5bp) | hsa-miR-4472 MIMAT0018999 Homo sapiens miR-4472 Targets (miRBase)(0.744) More Information \| Similar Motifs Found | motif file (matrix) |
| 9 |  | 1e-40 | -9.284e+01 | 0.70% | 0.31% | 10.3bp (17.2bp) | hsa-miR-1273g MIMAT0020602 Homo sapiens miR-1273g Targets (miRBase)(0.698) More Information \| Similar Motifs Found | motif file (matrix) |
| 10 |  | 1e-35 | -8.242e+01 | 0.46% | 0.18% | 9.1bp (11.2bp) | hsa-miR-1272 MIMAT0005925 Homo sapiens miR-1272 Targets (miRBase)(0.688) More Information \| Similar Motifs Found | motif file (matrix) |
| 11 |  | 1e-34 | -7.906e+01 | 0.61% | 0.28% | 10.5bp (12.2bp) | hsa-miR-126\* MIMAT0000444 Homo sapiens miR-126\* Targets (miRBase)(0.870) More Information \| Similar Motifs Found | motif file (matrix) |
| 12 |  | 1e-30 | -7.120e+01 | 9.06% | 7.67% | 10.8bp (12.4bp) | hsa-miR-4288 MIMAT0016918 Homo sapiens miR-4288 Targets (miRBase)(0.746) More Information \| Similar Motifs Found | motif file (matrix) |
| 13 |  | 1e-19 | -4.429e+01 | 0.66% | 0.38% | 9.0bp (15.2bp) | hsa-miR-568 MIMAT0003232 Homo sapiens miR-568 Targets (miRBase)(0.675) More Information \| Similar Motifs Found | motif file (matrix) |
| 14 |  | 1e-14 | -3.256e+01 | 0.78% | 0.52% | 10.1bp (14.8bp) | hsa-miR-4783-5p MIMAT0019946 Homo sapiens miR-4783-5p Targets (miRBase)(0.790) More Information \| Similar Motifs Found | motif file (matrix) |
